# A Mitochondria-Dependent Pathway Mediates the Apoptosis of GSE-Induced Yeast

**DOI:** 10.1371/journal.pone.0032943

**Published:** 2012-03-05

**Authors:** Sishuo Cao, Wentao Xu, Nan Zhang, Yan Wang, YunBo Luo, Xiaoyun He, Kunlun Huang

**Affiliations:** 1 Laboratory of food safety and molecular biology, College of Food Science and Nutritional Engineering, China Agricultural University, Beijing, People's Republic of China; 2 The Supervision, Inspection and Testing Center of Genetically Modified Organisms, Ministry of Agriculture, Beijing, People's Republic of China; Université Joseph Fourier, France

## Abstract

Grapefruit seed extract (GSE), which has powerful anti-fungal activity, can induce apoptosis in *S. cerevisiae*. The yeast cells underwent apoptosis as determined by testing for apoptotic markers of DNA cleavage and typical chromatin condensation by Terminal Deoxynucleotidyl Transferase–mediated dUTP Nick End Labeling (TUNEL) and 4,6′-diaminidino-2-phenylindole (DAPI) staining and electron microscopy. The changes of ΔΨmt (mitochondrial transmembrane potential) and ROS (reactive oxygen species) indicated that the mitochondria took part in the apoptotic process. Changes in this process detected by metabonomics and proteomics revealed that the yeast cells tenaciously resisted adversity. Proteins related to redox, cellular structure, membrane, energy and DNA repair were significantly increased. In this study, the relative changes in the levels of proteins and metabolites showed the tenacious resistance of yeast cells. However, GSE induced apoptosis in the yeast cells by destruction of the mitochondrial 60 S ribosomal protein, L14-A, and prevented the conversion of pantothenic acid to coenzyme A (CoA). The relationship between the proteins and metabolites was analyzed by orthogonal projections to latent structures (OPLS). We found that the changes of the metabolites and the protein changes had relevant consistency.

## Introduction

Grapefruit seed extract (GSE), also known as citrus seed extract, is a commercial product derived from the seeds and pulp of grapefruit. Chemical research revealed that it contains large quantities of polyphenolic compounds, such as catechins, epicatechin, epicatechin-3-O-gallate and dimeric, trimeric and tetrameric procyanidins [1]. GSE is commonly reported to have powerful antibacterial, antiviral and anti-fungal properties [2]–[5]. Thus, GSE could be used to protect vegetables and fruits from microorganisms [6]–[7]. GSE is an effective fungicide [4] and Ionescu et al. [2] demonstrated that GSE performed as well as other antimicrobial agents tested on 93 strains of fungus. As anti-fungal mechanism of GSE wasn't revealed, yeast cells, an ideal model organism to investigate various aspects of eukaryotes biology, was employed to study the apoptosis induced by GSE to disclose the anti-fungal mechanism.

Cell death can be divided into two main categories: necrosis and apoptosis [8]. Cells die in response to a variety of stimuli and during apoptosis they do so in a controlled, regulated fashion. This makes apoptosis distinct from another form of cell death called necrosis in which uncontrolled cell death leads to rapid death without process. Apoptosis, by contrast, is a process in which cells play an active role in their own death (which is why apoptosis is often referred to as cell suicide) [9]–[10]. In mammalian cells, two major apoptotic pathways have been described. One requires the participation of the mitochondria and is called the “intrinsic pathway”, and the other one, in which mitochondria are bypassed and caspases are activated directly, is called the “extrinsic pathway” [11]–[12]. For the mitochondrial pathway, certain main events have been regarded as integral control elements in the cell's decision to die, namely, the elevation of mitochondrial transmembrane potential (ΔΨmt) and the production of reactive oxygen species (ROS) [13]–[15]. Here the anti-fungal mechanism of GSE induced apoptosis was studied in yeast cells, which is an ideal model organism to investigate various aspects of mitochondrial biology [16].

Laun and colleagues used global transcriptome analysis to investigate the mechanism underlying the apoptotic phenotype of *S. cerevisiae* using temperature-sensitive Dcdc48^S565G^ [17] or Dorc2-1 [18] cells. The genes involved in cell-cycle regulation, DNA repair, oxidative stress response, mitochondrial functions and cell-surface rearrangement were differentially regulated during yeast apoptosis [19]. However, in mammalian cells, a large fraction of the events guiding cell death programs are dependent on protein post-translational modification rather than on genomic regulatory pathways [20]. Therefore, the functional characterization of proteins and regulatory networks involved in these processes is essential to further elucidate apoptosis as a mechanistic phenomenon. Metabolic fluxes constitute a fundamental determination of cell physiology because they provide a measure of the degree of engagement of various pathways in overall cellular functions and metabolic processes. Metabonomics and proteomics were firstly used together to investigate the yeast apoptosis mediated by mitochondria-dependent pathway. In the present article, we provided evidence that GSE-induced cell death exhibited features in common with apoptosis. Metabonomics and proteomics analysis revealed that the cells initially resisted dying and why they eventually, and irreversibly, committed suicide.

## Materials and Methods

### Yeast strains and media

The yeast *S. cerevisiae* strain W303-1A was used in this study. The cells were grown in YPD medium containing 1% yeast extract, 2% tryptone and 2% glucose. The growth experiments were performed in 250-mL flasks at a 2∶1 ratio of air-to-liquid and incubated on a mechanical shaker (200 rpm) at 28°C. For experiments, cells were inoculated from an overnight culture to fresh medium at OD_600_ = 0.1, and then incubated at 28°C with shaking before specific assays.

### Susceptibility test

Susceptibility to GSE (Bio/Chem Research, USA) is expressed as the minimum inhibitory concentration (MIC) to inhibit 80–90% of the growth of *S. cerevisiae*. Stationary phase cells were harvested and suspended (10^7^–10^8^ cells/mL) in YPD medium containing 0 mg/mL, 0.03 mg/mL, 0.06 mg/mL, 0.13 mg/mL, 0.25 mg/mL, 0.5 mg/mL and 1 mg/mL GSE. Following an incubation period of 3 h at 28°C with shaking (200 rpm), the bottles were examined for growth. After 3 h, the cells were serially diluted with sterile deionized water from 10^−4^ to 10^−6^ and spread onto YPD plates. The number of colony forming units (cfu) was determined after incubation at 28°C for 72 h. The cell count (cfu) of the GSE-treated cells were compared with those just before GSE treatment and expressed as a survival ratio (cfu %). The survival ratios were expressed as the average values with a standard deviation (STDEV) of at least three independent experiments [21].

### Test for apoptotic markers

For the *in vivo* staining of the nuclei, the cells were washed twice with PBS and treated with the 4,6′-diaminidino-2-phenylindole (DAPI) (Sigma, USA) dye at a final concentration of 2.5 µg/mL. Then, the cells were protected from light for 15 min and washed 6 times with PBS before microscopic observation [22]. For image acquisition, we used a BX 51 OLYMPUS fluorescence microscope (OLYMPUS, Japan) with excitation and emission settings of 358 and 461 nm, respectively. The DNA strand breaks were detected using a terminal deoxynucleotidyl transferase-mediated nick end labeling (TUNEL) kit (Roche, USA) according to the manufacturer's instructions. The slides were observed under visible light.

### Electron microscopy

The yeast cells were fixed with phosphate-buffered glutaraldehyde. After removing the cell walls, the cells were postfixed with osmium tetroxide and uranyl acetate and then dehydrated according to previously published methods for stationary phase cells [23]. After washing with 100% ethanol, the cells were washed with 100% acetone and infiltrated with 50% acetone/50% Epon for 30 min and 100% Epon for 20 h. The cells were transferred to fresh 100% Epon and incubated at 56°C for 48 h before thin sections were cut and stained with lead acetate [16]. The cell images were acquired using a transmission electron microscope (JEM-1230, JEOL, Japan).

### Mitochondrial transmembrane potential (ΔΨmt) assay

The mitochondrial transmembrane potential (ΔΨ) was estimated in cells treated with the laser dye rhodamine 123 (Sigma, USA). The cells were washed twice with PBS and treated with rhodamine 123 at a final concentration of 10 µg/mL. The cells were shaken at 100 rpm for 30 min in the dark at 28°C [24]. After incubation, the cells were washed 6 times with PBS before FACS analysis using a BD FACSCalibur equipped with FL1 (BD Biosciences, USA).

### Detection of reactive oxygen species

For ROS detection, GSE-treated cells were harvested by centrifugation at 5000 rpm for 5 min, washed once with PBS, and resuspended in 10 µg/mL 2′,7′-dichlorodihydrofluorescein diacetate (DCFH-DA) (Sigma, USA) dissolved in PBS from a stock solution of 2.5 mg/mL in ethanol. The concentration of the cells was approximately 5×10^6^ cells/mL, and the cells were incubated for 2 h at 28°C in the dark [21]. The cells were analyzed using a BD FACSCalibur at a high flow rate with excitation and emission settings of 488 and 525–550 nm (filter FL1), respectively.

### Metabonomics analysis

#### Metabolite extraction

50-mL cultures were cooled on ice, pelleted by centrifugation (5 min, 4°C, 3000 g), washed twice in ice-cold water, and extracted by a method modified from Bundy et al. [25]. Briefly, 5 mL of 75% ethanol (v/v) at 80°C was added directly to the cell pellet together with 2 mL of 0.3 mm diameter glass beads. The mixture was vortexed for 30 sec, heated to 80°C for 3 min and vortexed again for 30 sec. The supernatant was decanted, and the beads were washed with an additional 2 mL of 75% ethanol (v/v). The extract was combined with the wash, centrifuged (10 min, 16,000 g) to remove cell debris and then dried in a rotary vacuum concentrator at 30°C.

#### NMR spectroscopy

The dried cell extracts were dissolved in 0.8 mL of buffer (0.1 M phosphate buffer at pH 7.0, in ^2^H_2_O, containing 1 mM sodium trimethylsilyl-2,2,3,3-tetradeuteroproprionate; TSP) and then passed through 0.22-µm filters. The filtrate (0.6 mL) was transferred to a 5-mm NMR tube and analyzed using a Varian INOVA spectrometer interfaced to a 14.1 T magnet, yielding a resonance frequency for ^1^H of 600 MHz. One-dimensional spectra were acquired across a spectral width of 8 kHz into 32 K data points using the NOESY pulse sequence: Relaxation Delay (RD) = 2 sec. Water signals were suppressed by presaturation. Typically, 128 transients were acquired per sample. Spectral processing was carried out using an ACD/Labs NMR Processor v12.0. The chemical shifts were referenced to the TSP resonance (δ = 0.0) [25].

### Proteomic analysis

#### Protein extraction

The extraction of total proteins was performed as described by Almeida et al. with some modifications [26]. All procedures described below were carried out at 4°C. Briefly, 0.2 g of yeast powder were ground in liquid nitrogen and then homogenized in 1 mL of homogenization buffer containing 20 mM Tris-HCl (pH 7.5), 250 mM sucrose, 10 mM EGTA, 1 mM phenylmethanesulfonyl fluoride (PMSF), 1 mM dithiothreitol (DTT) and 1% Triton X-100. The homogenate was centrifuged at 20,000 *g* for 20 min, and the supernatant was removed to a new tube. The proteins in the supernatant were precipitated with 10% TCA on ice for 60 min and then centrifuged at 15,000 *g* for 10 min. The pellet was washed with acetone (twice with 10 mL and once with 1 mL) and then solubilized in 500 µL of lysis buffer containing 7 M urea, 2 M thiourea, 4% (v/v) CHAPS, 1% (w/v) DTT (Sigma, USA), 1% (v/v) IPG buffer pH 4–7, 1% (v/v) IPG buffer pH 3–10 NL (GE) and 0.5% (v/v) protease inhibitor cocktail. The protein concentration was quantified with a 2-D Quant kit (GE Healthcare, USA) using bovine serum albumin as the standard. The protein samples were stored at −80°C prior to use.

### 2-Dimensional electrophoresis

The 2-dimensional electrophoresis of protein extracts was performed using a GE Healthcare 2-DGE system according to the manufacturer's manual. Briefly, protein samples (400 µg) were mixed in 250 µL of rehydration buffer (7 M urea, 2 M thiourea, 4% (w/v) CHAPS, 2% (w/v) IPG buffer (pH 3–10) (GE Healthcare, USA) and 0.002% bromophenol blue). The samples were loaded onto IPG strips (13 cm, with a nonlinear range of IPG pH 3–10 NL; GE Healthcare) after brief sonication and centrifugation. Electrofocusing was carried out in an IPGphor isoelectric focusing system at 20°C for 20 kVh according to the manufacturer's instructions. Prior to the second 2-dimensional electrophoresis step, IPG strips were equilibrated by reduction with DTT and carboxymethylation with iodoacetamide. The equilibrated strips were run on 12.5% SDS polyacrylamide gels at 10 mA/gel for 1 h and 20 mA/gel until the dye front (bromophenol blue added in the agarose solution) reached the bottom end of the gel. The proteins were visualized with Coomassie Brilliant Blue R-250 after protein fixation for 1 h in a solution made of 50% ethanol, 10% acetic acid and 40% water. Destaining was performed with a solution made of 30% ethanol, 8% acetic acid and 62% water for 2 h, followed by five washes with water.

### Image analysis of 2D gels

**Figure 1:**
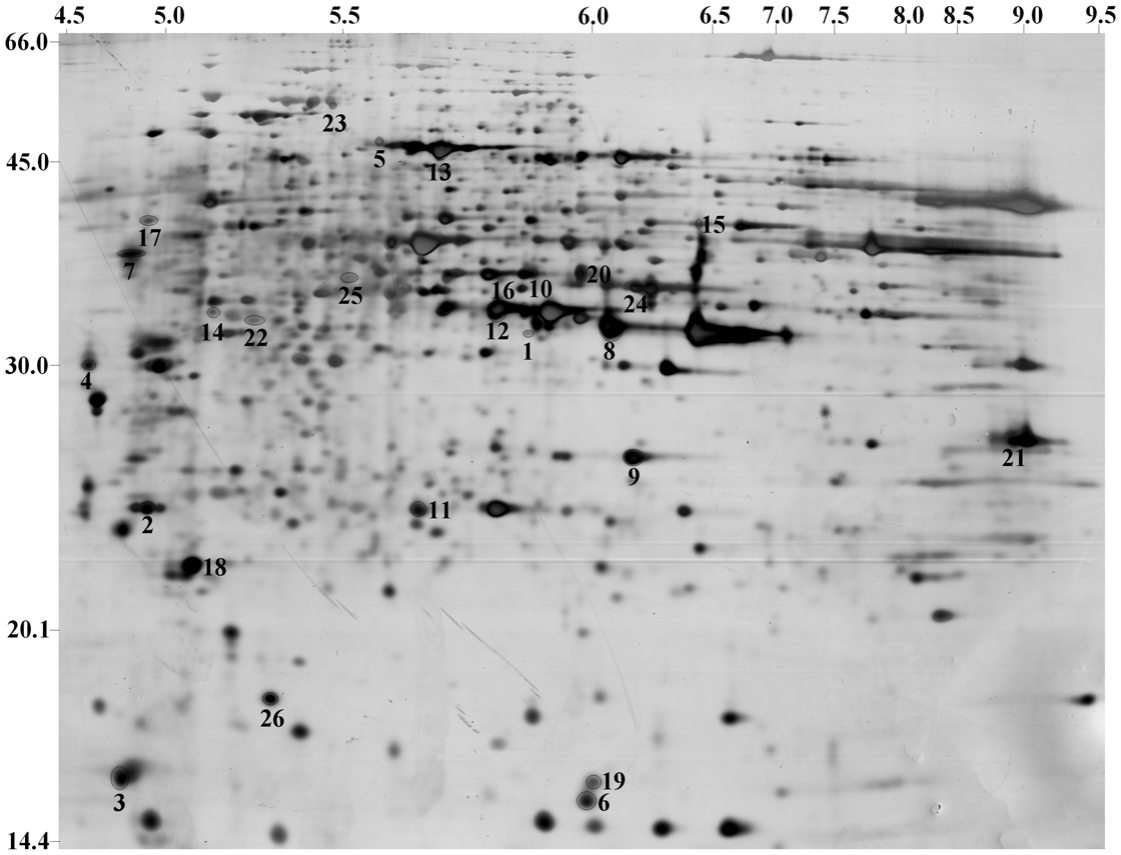
Representative silver-stained 2-D gel of total cellular extracts from yeast cells treated with GSE. The spots that were altered upon treatment are represented by numbers (1–26).

Image digitization was carried out with an Image Scanner (GE Healthcare, USA) in transmission mode. The comparison of the protein expression in the 2D gel images was performed using Image Master 2D Elite software (GE Healthcare, USA). To account for experimental variation, triplicate gels, from protein extracts obtained from independent experiments, were analyzed for each treatment. Spot detection was carried out automatically, and those spots showing faint intensity near the detection limit of colloidal CBB were not included in the comparisons. Prior to the automatic matching of spots between gel images, one gel was selected as the reference gel for each treatment. The amount of a protein spot was calculated based on the volume of that spot. To reflect the quantitative variations in intensity of protein spots between the control and treated samples, the spot volume was normalized as a percentage of the total volume of all the spots on the corresponding gel. Statistical analysis of the data was performed using SPSS software version 11.5 (SPSS Inc., Chicago, IL). The normalized intensity of spots on three replicate 2D gels was averaged, and the standard deviation was calculated for each treatment. A two-tailed non-paired Student's t test was used to determine whether the relative change was statistically significant between control and GSE-treated samples. Only spots that changed significantly in averaged normalized spot volume were excised for protein identification (Figure 1).

### In-gel tryptic digestion and protein identification by MS/MS

Protein spots with significant changes (at least two-fold) were carefully cut from Coomassie Brilliant Blue R-250-stained gels and subjected to in-gel trypsin digestion according to Sun et al., with minor modifications [27]. Gel pieces were first discolored in 50% (v/v) acetonitrile (ACN) and 25 mM ammonium bicarbonate and then subjected to reduction and alkylation with 10 mM of DTT and 55 mM of iodoacetic acid, respectively. Following vacuum drying, the gel pieces were incubated for 16 h at 37°C with sequencing-grade modified trypsin (Promega, USA) at a final concentration of 0.01 mg/mL in 25 mM ammonium bicarbonate. The supernatants were collected, vacuum-dried, and then redissolved in 50% ACN and 0.1% (v/v) trifluoroacetic acid (TFA) for MS analysis.

MALDI-TOF/TOF MS/MS experiments were carried out according to Zhang et al., with minor modifications [28]. The GPS Explorer™ software version 3.6 (Applied Biosystems) was used to create and search files with the MASCOT search program for peptide and protein identification. The NCBI Yeast Database 2011 was used for the search and was restricted to tryptic peptides. Carboxymethyl and oxidation were selected as variable modifications. One missed cleavage was permitted. The precursor error tolerance was set to ∼0.2 Da, and the MS/MS fragment error tolerance was set to ∼0.3 Da. All of the proteins identified had protein scores greater than 61 and individual ion scores greater than 21, with expected p values less than 0.05. All of the MS/MS spectra were further validated manually. In cases where multiple significant hits were found for a protein, only the highest scoring hit was listed in Table 1.

**Table 1:** The MS/MS analysis of intracellular proteins showing differential expression under GSE stress.

Spot ID	Ratio	NCBI accession	Protein	Molecular function	Protein score	pI/MW (experimental)	pI/MW (theoretical)
Protein synthesis							
1	34.51	gi|126663628	RPS6A	40 S ribosomal protein S6 (S10) (YS4) (RP9)	91	5.96/32.5	9.08/50.2
2	2.47	gi|254565973	YBL0613	40 S ribosomal protein S8 (S14) (YS9) (RP19)	80	4.83/24.7	10.28/22.6
3	−1.67	gi|254572585	RPL14A	60 S ribosomal protein L14-A	132	4.67/16.1	4.77/14.9
4	3.15	gi|254564587	RPP0	60 S acidic ribosomal protein P0 (A0) (L10E)	157	4.63/30.0	4.63/33.7
5	−7.33	gi|116293731	TEF1	Elongation factor 1-alpha	123	5.57/48.5	9.11/50.1
6	2.91	gi|254564995	CPR2	Peptidyl-prolyl cis-trans isomerase B precursor	263	5.97/16.1	6.06/18.1
Proteins related to energy							
7	8.83	gi|254565279	GDH1	NADP-specific glutamate dehydrogenase 1	85	4.82/38.5	7.72/49.6
8	1.89	gi|2494641	TDH1	Glyceraldehyde-3-phosphate dehydrogenase 1	221	6.08/33.3	6.54/35.6
9	2.86	gi|126131588	GPM1	Phosphoglycerate mutase 1	154	6.24/26.4	5.28/27.6
10	3.94	gi|254571987	FBP1	Fructose-1,6-bisphosphatase	77	5.95/38.5	6.12/38.4
11	2.37	gi|230405	TPI1	Triose phosphate isomerase	224	5.65/24.6	5.73/27.1
12	5.26	gi|254568544	ADH1	Alcohol dehydrogenase 1	350	5.81/34.3	5.84/37.3
13	6.23	gi|254570575	PDC1	Pyruvate decarboxylase isozyme 1	86	5.69/47.3	5.64/61.5
14	−6.86	gi|84873873	PDB1	Pyruvate dehydrogenase E1 component subunit beta, mitochondrial precursor	222	5.13/33.9	5.07/39.6
15	−3.28	gi|496718	CIT1	Citrate synthase, mitochondrial precursor	119	6.43/40.5	7.81/51.9
16	8.38	gi|150951593	MDH1	Malate dehydrogenase, mitochondrial precursor	175	5.95/34.2	6.46/34.7
17	2.09	gi|6322581	ATP2	ATP synthase subunit beta, mitochondrial precursor	106	4.83/40.7	5.52/54.8
Redox protein							
18	3.53	gi|146418172	TSA1	Peroxiredoxin	253	5.08/22.6	4.96/21.7
19	UP	gi|50552880	SOD1	Superoxide dismutase [Cu-Zn]	84	5.97/15.9	5.93/15.8
Membrane protein							
20	46.93	gi|7331158	MPG1	Mannose-1-phosphate guanyl transferase	104	5.97/36.8	6.04/40.0
21	2.04	gi|254569372	POR1	Outer mitochondrial membrane protein porin 1	291	8.93/27.2	9.02/29.6
22	46.92	gi|218716961	LSP1	Sphingolipid long chain base-responsive protein	109	5.15/32.8	5.45/39.4
23	27.36	gi|119489725	RHO1	GTP-binding protein	73	5.46/56.5	7.63/37.7
DNA repair protein							
24	UP	gi|254571131	TAH18	Probable NADPH reductase	121	6.19/38.6	6.03/39.2
25	24.36	gi|133901976	RAD51	DNA repair protein	74	5.03/36.5	6.17/65.5
Cell structure-related							
26	3.64	gi|254566063	COF1	Actin-depolymerizing factor 1	281	5.29/18.3	6.14/18.2

### Multivariate Data Analysis

Each ^1^H NMR spectrum from cell extracts was segmented into 217 integrated regions of equal width (0.04 ppm), corresponding to the region δ0.0–10.0 using ACD/Labs NMR Processor v12.0. The area for each segmented region was calculated, and the integral values contributed to an intensity distribution of the whole spectrum. The region (δ4.5–6.0) was excluded prior to statistical analysis to remove the variation in water suppression efficiency. All remaining regions of the spectra were scaled to the total integrated area of the spectra to reduce any significant concentration differences. The data set was mean centered prior to PCA and PLS-DA processing [29].

First, to discern the presence of inherent similarities of spectral profiles, an unsupervised pattern recognition (PR) method, PCA, was conducted for the cell extracts samples (SIMCA-P+_12.0). Next, a supervised PR method, PLS-DA, was performed to maximize the separation between the changes observed in the cell extracts samples [30]. In PLS-DA, the *X* matrix is a measured matrix, i.e., NMR data, and the *Y* matrix is made of dummy variables consisting of ones and zeros that indicate the class for each treatment [31]–[32]. The OSC and Pareto were used before the PCA and PLS-DA. VIP (variable influence on projection) values characterize the relative overall importance of the individual X variables to the model, and the weights for each component of the model reveal the nature of the relationship between each X variable and the predicted quantity Y.

The ^1^H NMR and proteomic data were integrated by OPLS methods, where the proteomic data were used for modeling and prediction of ^1^H NMR data by using a model with one predictive component and one ^1^H NMR-orthogonal component (data were mean-centered and scaled to unit variance before modeling). The predictive performance was evaluated by five-fold cross-validation, where the Q^2^ value (goodness of prediction) was used to select regions of the ^1^H NMR spectra of interest (Q^2^>0.4). The ^1^H NMR peaks (cell extracts metabolites) related to the proteins were determined by interpretation of each respective OPLS model [33].

## Results

### GSE-induced yeast cell death has features in common with apoptosis

The anti-fungal activity of GSE on *S. cerevisiae* was tested in YPD. The addition of increasing levels of GSE reduced growth yield. An addition of 0.03 mg/mL GSE had little or no effect on the growth, while the first notable inhibitory effect was observed with the addition of 0.06 mg/mL GSE. The MIC was defined as 0.13 mg/mL because this condition was deemed appropriate for our study (data not shown) and were used for all subsequent analyses unless otherwise indicated.

**Figure 2:**
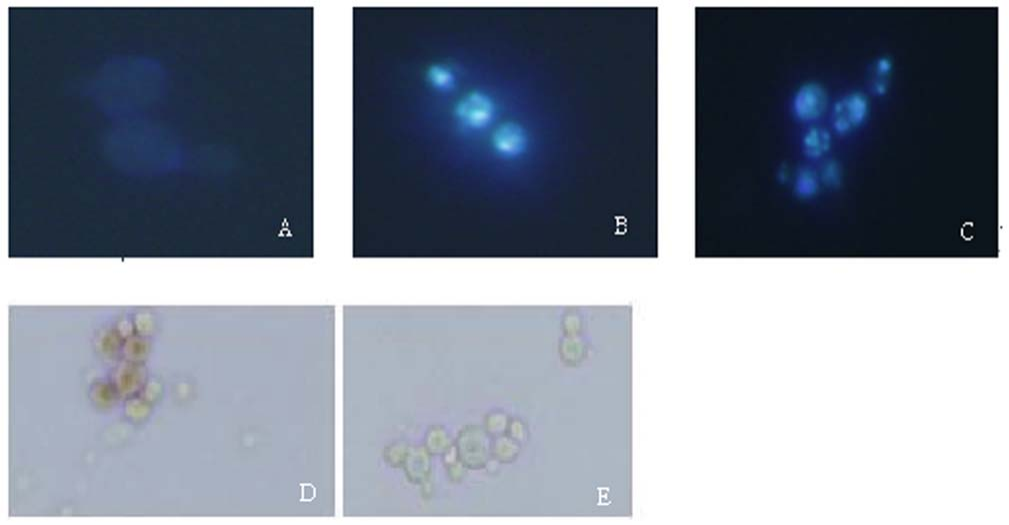
DAPI- and TUNEL-stained *S. cerevisiae* nuclei (10×60). A, E, control group; B, C, D, S. cerevisiae treated with 0.13 mg/mL of GSE for 3 h; A–C yeast cells stained with DAPI; D and E yeast cells stained with TUNEL.

To determine whether the GSE-induced cell death of yeast is consistent with apoptosis, we examined the morphology of the chromatin after treatment with GSE. As shown in Figure 2, we found that, like other cell types undergoing apoptosis, GSE-treated cells had distinct chromatin condensation (Figure 2B) coincident with nucleus fragmentation (Figure 2C). The nuclei of the untreated cells were homogeneous in shape and density (Figure 2A). Apoptotic stimuli have also been reported to cause DNA cleavage in the DNA. Therefore, we next tested whether GSE-induced yeast cells death was associated with DNA cleavage, which could be revealed by TUNEL staining. We observed that incubation with GSE for 3 h produced a strong TUNEL-positive phenotype in approximately 50% of the cells (Figure 2D), indicating massive DNA fragmentation. The control group showed no TUNEL-positive phenotype (Figure 2E).

### Electron microscopy

**Figure 3:**
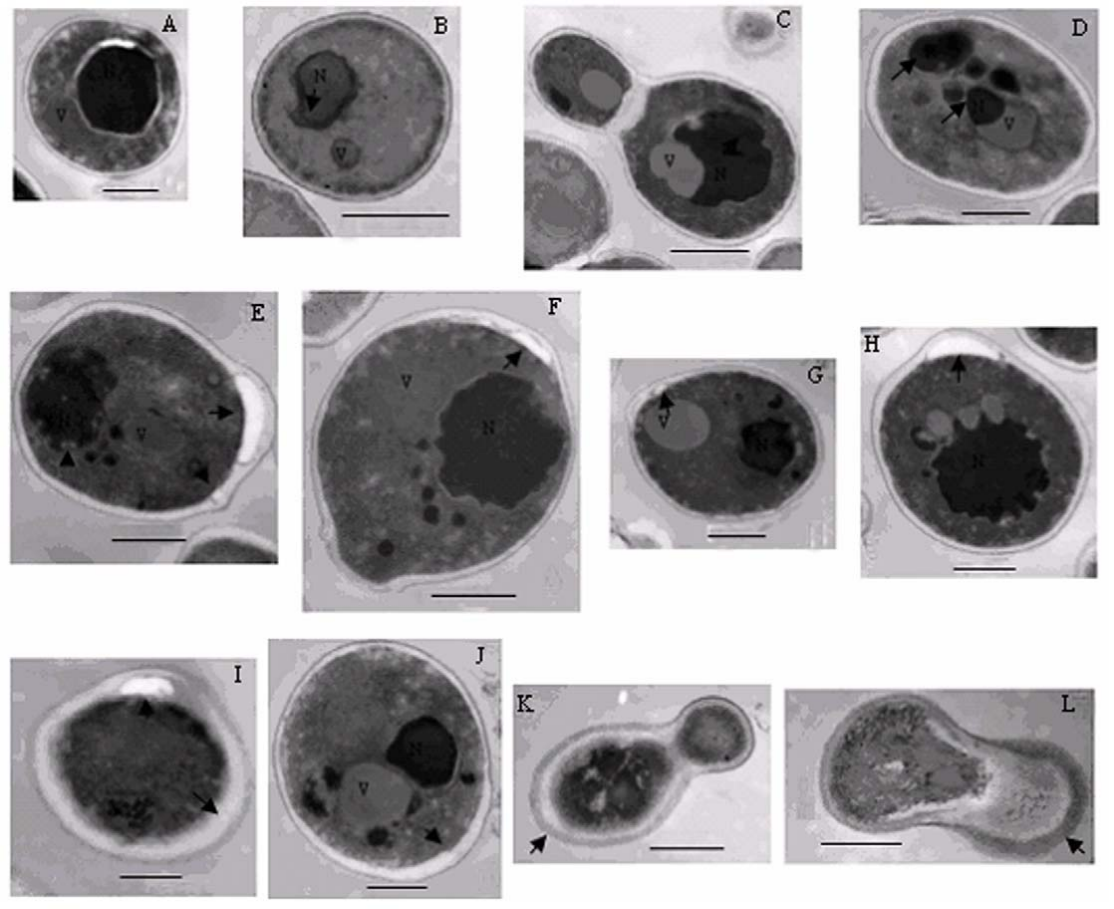
Electron microscopy images of *S. cerevisiae*. L, control group; A–K, S. cerevisiae treated with 0.13 mg/mL of GSE for 3 h. Bar, A, C, 1 µm; B, D–L, 500 nm. Arrows show the changes mentioned in the paper.

The exposure of the *S. cerevisiae* cells to GSE induced many of the morphological characteristics of apoptosis. The nuclei of untreated cells showed normal cellular morphology with a distinct cell wall, an intact nucleus and numerous membranous organelles (Figure 3A). In contrast, electron microscopic images of cells incubated with 0.13 mg/mL GSE revealed extensive chromatin condensation (Figure 3B, 3C), multiple nuclear fragments (Figure 3D), nuclear vesicle formation (Figure 3E), and irregular structures protruding from the nucleus (Figure 3F); these features are consistent with the induction of apoptosis in cells. In addition, some cells showed tiny vesicles on the outer side of the plasma membrane (Figure 3E, 3F, 3G, 3H, 3I). This could be an equivalent of membrane blebbing which is a characteristic marker of apoptosis. Some cells showed distended cell wall, ruptured internal organelles and the withdrawal of the cytoplasmic membrane from within the cell wall (Figure 3J), the cytoplasmic membrane even can disappear totally (Figure 3I, 3K) and cell plasm exudation (Figure 3L).

### GSE-induced apoptosis is accompanied by mitochondrial alterations

**Figure 4:**
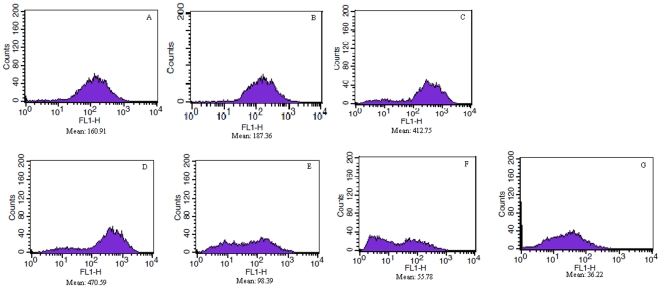
*S. cerevisiae* mitochondrial membrane potential (DYmt). A–G, S. cerevisiae treated with 0.13 mg/mL of GSE for 0, 0.5, 1, 1.5, 2, 2.5, and 3 h.

GSE treatment induced a transient mitochondrial membrane potential hyperpolarization followed by a depolarization. The mitochondrial membrane potential (ΔΨmt) was stable and only had a little growth after 0 to 0.5 h incubation with GSE. After incubation for 1 h, the ΔΨmt increased rapidly and reached a peak at 1.5 h. Subsequently, the ΔΨmt had a rapid decline and tended stabilize after 3 h. At last, although with a low ΔΨmt, the cells maintained the specific mitochondria staining indicating that the mitochondria membrane integrity was still preserved (Figure 4).

**Figure 5:**
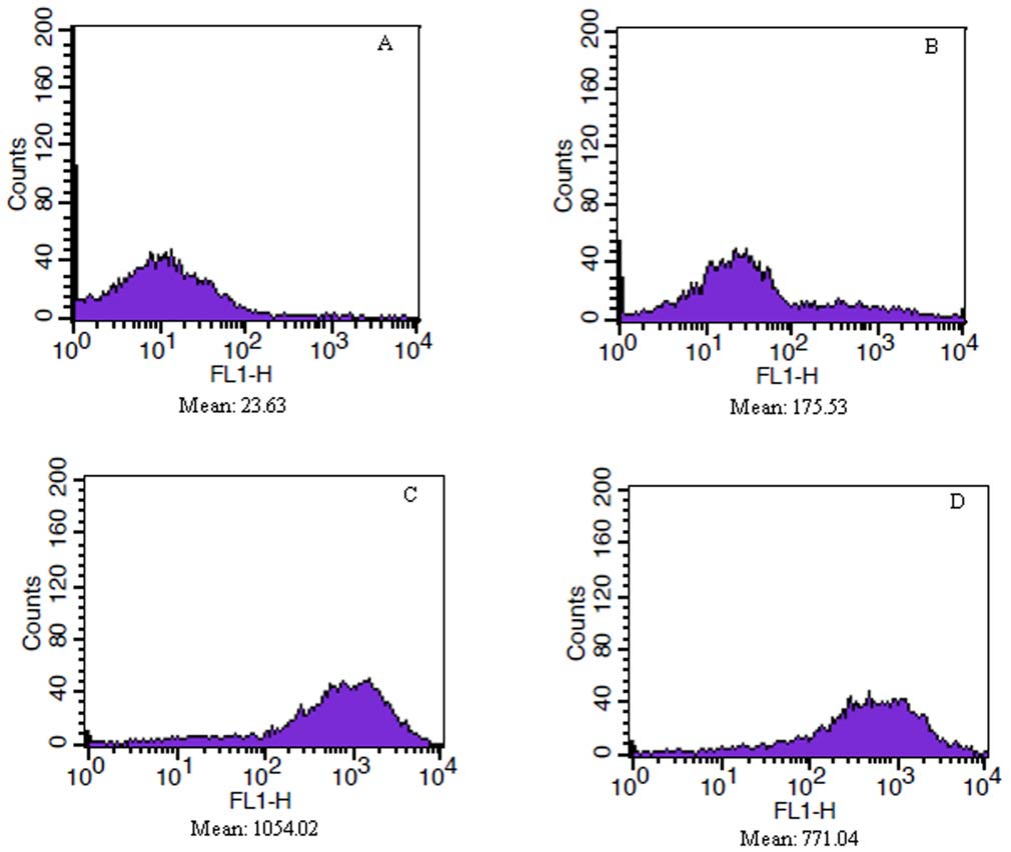
*S. cerevisiae* intracellular ROS. A–D, S. cerevisiae treated with 0.13 mg/mL of GSE for 0 h, 1 h, 2 h, and 3 h.

The cells were treated with or without 0.13 mg/mL GSE for 0–3 h, mixed with the ROS indicator DCFH-DA and subjected to FACS analysis (Figure 5). The cells that were not treated with GSE showed low intracellular ROS levels, but GSE-treated cells showed a significant increase in the amount of ROS after treatment with GSE for 2 h. The ROS levels declined slightly after 3 h.

In summary, the treatment of yeast cells with GSE leaded to hyperpolarization of the mitochondrial membrane potential ΔΨmt. The elevation of ΔΨmt promoted ROS production. As evidenced above, we have shown that the mitochondria-dependent pathway mediated GSE-induced apoptosis.

### Metabonomics analysis by ^1^H NMR

**Figure 6:**
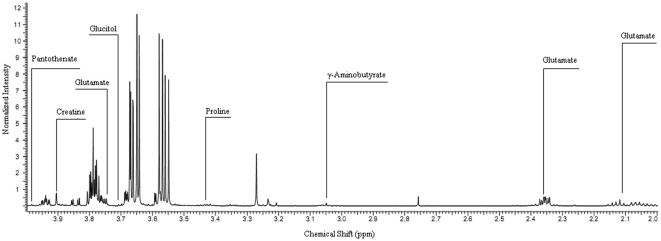
1H NMR spectra of a typical cell extract.

Analysis of the PCA and PLS-DA (data not shown) highlighted the regions of the single-pulse ^1^H NMR spectra that were influential discriminators in the separation and classification of the GSE-treated and control samples (Figure 6). The GSE-treated cells exhibited lower concentrations of glutamate and γ-Aminobutyrate and higher concentrations of proline, creatine, glucitol and pantothenate (Table 2).

**Table 2:** The comparison of the intracellular metabolites of GSE-treated and control yeast cells.

Chemical shift(ppm)	Metabolites	GSE/Control
3.97	Pantothenate	1.86
2.04, 2.09, 2.33, 3.74	Glutamate	−2.56
3.03	γ-Aminobutyrate	−3.72
3.87	Creatine	1.31
3.72	Glucitol	2.34
3.40	Proline	1.65

### Proteomic analysis by 2D and MS

The expression of the ribosomal proteins RPS6A, YBL0613 and RPP0 was increased. However, RPL14A expression did not increase, and TEF1 expression was decreased. The expression of the peptidyl-prolyl cis-trans isomerase CPR2 was increased when the yeast cells were treated with GSE. The levels of most of the energy-related proteins were increased (GDH1, TDH1, GPM1, MDH1, TPI1, ADH1, PDC1, ATP2, FBP1), but the expression of the CIT1 and PDB1 proteins was decreased. Reducible enzymes, TSA1 and SOD1, were increased, which indicated that the yeast cells tried to maintain the redox process. During the stages of apoptosis, the membrane related proteins MPG1, POR1, LSP1 and RHO1 were increased, and DNA repair proteins, TAH18 and RAD51, were also increased. COF1 was increased to maintain cell shape and internal structure. Several times increased expression occurred for a number of proteins, which were related to membrane stability and DNA repair.

### Correlation patterns between ^1^H NMR and proteomic data

The correlation between the ^1^H NMR and proteomic data was analyzed by OPLS model. The results showed that the DNA repair proteins, TSA1, SOD1, COF1, RPS6A, POR1, MPG1, LSP1, RHO1, GDH1, TPI1, MDH1, CIT1, ATP2 and TAH18, were all associated with glutamate; ATP2 was also related to creatine.

## Discussion

GSE is a natural rich source of the polyphenols. The polyphenols in the GSE were often measured as gallic acid equivalent [34]. Gallic acid was identified as the major activity component of GSE in many articles [35]–[37]. GSE exerted potent antifungal activity against the yeast-like fungi strains and lower activity against dermatophytes and molds [38]–[39]. In another experiment Ignacio and Thai demonstrated that GSE is as effective as miconazole nitrate salt [40]. In this study we found 0.13 mg/mL GSE could inhibit the yeast effectively, which is coincident with [40]. As no one had reported why GSE could kill the fungi. Then it is helpful to study the inhibitory mechanism for better understanding of this fungicide.

Severin and colleagues elegantly extended their findings towards a timeline of events, proposing a scheme of the mitochondrial death cascade in yeast. In summary, treatment of yeast cells with α-factor or amiodarone leads to hyperpolarisation of the mitochondrial membrane potential ΔΨmt. Elevation of ΔΨmt promotes ROS production, which then initiates the mitochondrial thread-grain transition (mitochondrial fragmentation) and de-energisation. The mitochondrial de-energisation finally results in loss of ΔΨmt [41], [42]. ΔΨmt and ROS changes in our study were consistent with Pozniakovsky et al. and Severin et al. [41], [42].

Proline is a water-soluble amino acid that prevents cell death from dehydration under osmotic stress [43] and increased the performance of yeast against GSE. The pantothenic acid content increased because its conversion to CoA was blocked. Without CoA, pyruvic acid could not enter into the citric acid cycle, which was consistent with reduction of CIT1 and PDB1. Although the synthesis of CoA was not effective, dissipative γ-Aminobutyrate turned into succinate which could enter into the TCA cycle to support the energy generation during the apoptosis [44]–[46]. The increase of creatine illustrated that the yeast cells mainly relied on aerobic respiration under the GSE suppressed condition [47]. The increase in glucitol was a sign of the decomposition of the cell wall [48].

GSE reduced the expression of RPL14A. We previously measured the total amount of the protein expression decreased in line and found that the expression of TEF1 was reduced. CPR2 expression increased due to protein structure distortion under the GSE stress.

Changes in glutamate and glutamate dehydrogenase expression were consistent. The expression of glutamate declined, while the expression of glutamate dehydrogenase increased. Glutamate could still produce energy despite the decline in citrate synthase because the glutamate-generated α-Ketoglutarate could continue to enter into the citric acid cycle to produce energy, which was consistent with the rise in malate dehydrogenase. Six glycolytic pathway proteins (TDH1, GPM1, FBP1, TPI1, ADH1 and PDC1) were increased, which explained the energy production during apoptosis that both occurs by glycolysis and glutamate metabolism in the TCA cycle. The increase of ATP2 showed that the production of energy was accelerated in yeast cells. Apoptosis was an energy-dependent process since apoptotic cells were metabolically active, and thus need high-energy compounds to maintain metabolic activity [49]. The results in this study are consistent with the above theory. GSE induced apoptosis changes of metabolites and proteins strengthen the energy production, although some part of the functions were damaged.

Cu/Zn superoxide dismutase (SOD1), a protein previously identified to have antioxidant properties, was upregulated with metals stress, indicating the protein may be actively involved in metal detoxification of yeast [50]. The results in this study is in good agreement with their work, because the stress proteins, TSA1 and SOD1, increased intracellularly to combat the explosive increase in oxidative substances. The increase of three kinds of cell wall proteins indicated that cell membrane damage had occurred and that the yeast cells were capable of remediation. Consistent with the report by Eisenberg et al. [51], the expression level of the membrane protein POR1 also increased. The increase in DNA repair enzymes demonstrated that yeast cells attempted to improve the DNA repair. The expression of COF1 increased, indicating the structural damage and repair of the cells.

Proteomics and metabonomics research is relative and based on the total content of the proteins and the metabolites in samples. GSE inhibition influenced protein expression and reduced the gross expression of metabolites in yeast. In this study, the relative increase of proteins and metabolites showed the tenacious resistance of the yeast cells to cell death, however, GSE induced yeast cell apoptosis by destruction of the mitochondrial 60 S ribosomal protein L14-A and prevention of the conversion of pantothenic acid to CoA.

The apoptotic phenotypes associated with CIT1 deletion were rescued by the addition of exogenous glutathione or glutamate, indicating that supply of glutamate for glutathione biosynthesis was likely to be a factor affecting ROS accumulation and cell death [52]. Glutamate can be converted to α-ketoglutarate, an integral component of the citric acid cycle. It is a component of the antioxidant glutathione. The cyclization of glutamate produces proline, an amino acid important for resisting apoptosis [53]. Although the role of glutamate was diminished in this study, it contributed substantially to fighting against apoptosis.

In summary, GSE-induced apoptosis was mediated by mitochondria-dependent pathway. Although initiated by the mitochondria, other organelles were also involved in this apoptosis. The changes in the yeast cells during apoptosis were indicative of their struggle with cell death. However, the destruction of the mitochondrial 60 S ribosomal protein L14-A and the cessation of the conversion of pantothenic acid to CoA resulted in apoptosis. The method can be used as a preliminary study of apoptosis, to identify interest proteins or metabolites and then further detailed study is needed to find out the exact mechanism of apoptosis.
